# Omaveloxolone Suppresses Cell Growth and Causes Cell Cycle Arrest by Downregulating CDC20 Expression in Glioblastoma Cells Both In Vitro and In Vivo

**DOI:** 10.1111/jcmm.70607

**Published:** 2025-05-29

**Authors:** Kuan‐Ting Lee, Yi‐Chiang Hsu, Ann‐Shung Lieu, Chih‐Lung Lin, Tai‐Hsin Tsai

**Affiliations:** ^1^ Graduate Institutes of Medicine, College of Medicine Kaohsiung Medical University Kaohsiung Taiwan; ^2^ School of Medicine I‐Shou University Kaohsiung Taiwan; ^3^ Division of Neurosurgery, Department of Surgery Kaohsiung Medical University Hospital Kaohsiung Taiwan; ^4^ Department of Surgery, School of Medicine, College of Medicine Kaohsiung Medical University Kaohsiung Taiwan; ^5^ Department of Surgery Kaohsiung Municipal Siaogang Hospital Kaohsiung Taiwan

**Keywords:** CDC20, cell cycle, glioblastoma, Nrf2, omaveloxolone

## Abstract

Omaveloxolone is a synthetic oleanane triterpene with considerable antitumor activity. It induces human glioblastoma (GBM) cell death in vitro and in vivo, but the underlying mechanism remains to be determined. In this study, GBM cell lines (GBM8401 and U‐87 MG cells) were exposed to different concentrations of omaveloxolone (0, 600, 800 and 1000 nM). A cell viability assay was conducted using the PrestoBlue Cell Viability Reagent. Three‐dimensional microscopy revealed changes in cell morphology. Cell cycle, apoptosis and mitochondrial membrane potential were tested using flow cytometry. The expression levels of cell cycle‐related proteins and genes were determined through Western blotting and next‐generation sequencing, respectively. The results indicated that omaveloxolone had significant selective cytotoxicity against human GBM cells and suppressed the migration and invasion of these cancer cells. It also caused cell cycle arrest through the downregulation of cell cycle‐related genes, including cell division cycle 20 homologue (*CDC20*), as revealed by next‐generation sequencing. In a xenograft tumour model, omaveloxolone decreased tumour volume and CDC20 expression. Taken together, these findings suggest that omaveloxolone is a potential drug candidate for GBM treatment by promoting GBM cell death through the downregulation of CDC20 expression.

## 1 Introduction

Glioblastoma (GBM) is the most common and lethal primary brain cancer in adults [1, 2]. Its standard treatment in newly diagnosed adults includes surgery, radiotherapy and concomitant and adjuvant chemotherapy with temozolomide [2]. Despite advancements in treatment, the long‐term survival of patients with GBM remains very low, with 2‐ and 5‐year survival rates of only 25% and 10%, respectively. Prognosis also remains poor due to resistance to chemotherapy and radiotherapy [1, 3]. GBM is a highly heterogeneous disease typically viewed from a pathological perspective. Its dynamic heterogeneity should be analysed at the cellular level to determine the origin of cells, identify potential therapeutic targets and detect the source of tumour recurrence [4, 5]. Therefore, the molecular mechanisms of GBM should be explored, and new therapeutic methods should be developed for the effective treatment of GBM in the future.

Anaphase‐promoting complex (APC) plays a crucial role in promoting the progression of the cell cycle through the M and G1 phases [6, 7]. APC and its coactivator, namely cell division cycle 20 homologue (CDC20), play a key role in mitotic transition [7]. Many studies have indicated that CDC20 is abnormally expressed in various types of solid cancer, with this abnormal expression linked to tumorigenesis and poor prognosis. CDC20 expression is upregulated in several types of cancer, including lung cancer [8], liver cancer [9], prostate cancer [10] and brain cancer [11]. Patients with higher expression levels of CDC20 exhibit shorter overall survival, indicating that the expression of CDC20 is an independent prognostic factor for many types of cancer [12, 13]. Targeting CDC20 expression inhibits the malignant progression of glioma; thus, CDC20 is regarded as an attractive target for glioma treatment [14, 15]. These findings indicate that CDC20 functions as an oncoprotein in various types of human cancer.

Omaveloxolone (2‐cyano‐3,12‐dioxooleana‐1,9‐dien‐28‐oicacid‐difluoro‐propylamide, CDDO‐DFPA, RTA 408) is a semisynthetic oleanane triterpenoid and a highly potent activator of nuclear factor erythroid 2‐like 2 (Nrf2) [16]. Nrf2 is a transcription factor that plays a dual role in cancer; specifically, it either exerts a cytoprotective effect or promotes cancer progression [17]. CDDO and its derivatives, including omaveloxolone, regulate cell growth through the following mechanisms. Omaveloxolone activates Nrf2, which translocates to the nucleus, where it binds with antioxidant response element (ARE) to initiate the expression of antioxidant enzymes and proteins, such as heme oxygenase‐1, NAD(P)H:quinone oxidoreductase‐1 and superoxide dismutase. Omaveloxolone inhibits the NF‐κB inflammatory pathway through the direct suppression of IKKβ kinase activity and the indirect detoxification of reactive oxygen species; this detoxification process is mediated by the activation of Nrf2 and the induction of cytoprotective gene transcription [16, 18]. Omaveloxolone also binds to KEAP1, resulting in the stabilisation and nuclear translocation of Nrf2, thereby promoting the expression of antioxidant response element (ARE)‐dependent genes, including HO‐1, NQO1 and SOD [19].

In addition to its antioxidant and anti‐inflammatory effects, omaveloxolone has demonstrated anticancer activities in several studies. It can inhibit cell proliferation, induce apoptosis and modulate cell cycle progression in multiple cancer models, including breast cancer, leukaemia, neuroblastoma and glioblastoma [20, 21]. Given its multifaceted biological activities, omaveloxolone is being actively investigated not only for neurodegenerative diseases, but also as a potential therapeutic agent for cancer treatment by targeting oxidative stress pathways and cell cycle regulators. However, NRF2 depletion also promotes cell cycle arrest by altering the levels of key cell cycle regulators [22]. Notably, omaveloxolone has been shown to induce G2/M cell cycle arrest and promote apoptosis by regulating the expression of key cell cycle proteins, such as cyclin B1, CDK1 and p21 [20]. However, limited data are available regarding how CDDO and its derivatives affect or regulate the cell cycle, and further research is required to determine the mechanisms underlying the anticancer effects of these drugs.

CDC20 is a highly conserved and essential regulator of the cell cycle and is considered a promising therapeutic target for various types of cancer [23]. CDC20 plays a key role in chromosome segregation and mitotic exit. It is also the target of the spindle assembly checkpoint and a key cofactor of APC or cyclosome (APC/C) E3 ubiquitin ligase. CDC20 regulates the activity of APC/C ubiquitin for specific substrates and leads to their subsequent degradation by proteasomes [24]. CDC20 also mediates the activation of APC and regulates cell cycle progression. Together with APC, CDC20 plays a crucial role in inhibiting or activating cell cycle regulatory factors such as CDKA and cyclin B, thereby controlling the transition from the metaphase to late cell division. Higher CDC20 expression has been observed in GBM than in low‐grade gliomas, with patients with higher CDC20 expression and GBM of the proneural subtype exhibiting significantly shorter overall survival [25].

In this study, omaveloxolone is an activator of Nrf2 that causes cell cycle arrest. Omaveloxolone may regulate or affect the expression of the CDC20 gene by activating Nrf2, thereby leading to cell cycle arrest and resulting in anticancer effects. In this study, we examined the anticancer effects of omaveloxolone, a novel synthetic oleanane triterpenoid, in human GBM cells both in vitro and in vivo and explored the mechanisms underlying these effects, suggesting that omaveloxolone may be a potential candidate for the treatment of GBM.

## 2 Materials and Methods

### 2.1 Preparation of Reagents and Chemicals

Omaveloxolone (RTA 408, CAS no. 1474034‐05‐3) was obtained from Cayman Chemicals. PBS, RPMI 1640 medium, Trypan Blue solution, dimethyl sulfoxide (DMSO) and trypsin–EDTA (0.25%) were purchased from Sigma‐Aldrich. Temozolomide (TMZ) powder (Sigma‐Aldrich, USA). Propidium iodide (PI) was obtained from Sigma‐Aldrich, and the markers were obtained from Bio‐Rad Laboratories. β‐Actin (1:20,000, Sigma, A5441), cyclin B1, cyclin D1 (1:1000, Proteintech, 55004‐1‐AP), CDK4 (1:1000, Sigma‐Aldrich, E1Z6R), CDK6 (1:1000, Cell Signalling Technology, D4S8S), p21 (1:1000, Cell Signalling Technology, E2R7A) and CDK1 (1:1000, Cell Signalling Technology, E1Z6R) antibodies were purchased from the corresponding manufacturers.

### 2.2 Cell Culture

The U‐87 MG glioblastoma cell line, originally derived from human astrocytoma, was obtained from the Food Industry Research and Development Institute (Hsinchu, Taiwan). Cells were maintained in minimum essential medium (MEM) supplemented with 10% fetal bovine serum (FBS; Gibco, USA) and 1% penicillin–streptomycin (P/S; Gibco, USA). The GBM8401 human glioblastoma cell line was purchased from the Bioresource Collection and Research Center (Hsinchu, Taiwan). GBM8401 cells were cultured in RPMI‐1640 medium (Gibco, USA) supplemented with 10% FBS and 1% P/S. Both cell lines were cultured in a humidified incubator at 37°C with 5% CO_2_. All culture media were supplemented with necessary nutrients and antibiotics to promote cell growth and prevent microbial contamination throughout the experiments.

### 2.3 Cell Viability Assay

Cell viability was assessed using the PrestoBlue Cell Viability Reagent (Invitrogen, USA), as previously described [20]. U‐87 MG and GBM8401 cells were seeded into 96‐well plates at a density of 3 × 10^3^ cells per well and incubated for 24 h at 37°C in a humidified atmosphere containing 5% CO_2_. Cells were then treated with omaveloxolone at concentrations of 0, 600, 800 or 1000 nM, or with vehicle (DMSO) for another 24 h. Cell viability was determined according to the manufacturer's instructions. Each treatment was performed in triplicate. The IC_50_ values were calculated based on cell viability measurements.

### 2.4 Colony‐Forming Assay

For the colony formation assay, GBM8401 and U‐87 MG cells were seeded into 6‐cm culture dishes at a density of 1 × 10^3^ cells per dish and incubated at 37°C with 5% CO_2_. After 24 h, cells were treated with omaveloxolone (0, 600, 800 or 1000 nM) for 24 h. The culture medium was refreshed every 3 days. After 14 days, colonies were fixed and stained with 0.5% crystal violet at room temperature for 30 min, and colony formation was assessed under a microscope.

### 2.5 Cell Cycle Assay

To evaluate the effect of omaveloxolone on cell cycle progression, a flow cytometry‐based assay was performed as previously described [20]. Briefly, 3 × 10^5^ cells were seeded in six‐well plates and incubated for 24 h. Cells were then treated with DMSO or various concentrations of omaveloxolone (0, 600, 800 and 1000 nM) for 24 h. Following treatment, cells were fixed in 70% ethanol at −20°C for at least 8 h. Fixed cells were washed with PBS and stained with a solution containing propidium iodide (PI), Triton X‐100 and RNase A for 30 min. DNA content was analysed using a FACSCalibur flow cytometer (BD Biosciences, USA), and data were processed with WinMDI 2.9 software.

### 2.6 Apoptosis Assay

To determine whether the negative effect of omaveloxolone observed on the viability of GBM8401 and U‐87 MG cells involved apoptosis, an apoptosis assay was conducted with an Annexin V‐FITC apoptosis dye. Briefly, GBM8401 and U‐87 MG cells were cultivated on six‐well culture plates (Orange Scientific, EU) and exposed to different concentrations of omaveloxolone (0, 600, 800 and 1000 nM) for 24 h. To remove the supernatant, the cell suspension was centrifuged at 1000 rpm for 3 min. Subsequently, the cells were rinsed with PBS and centrifuged at 1000 rpm for 3 min. After the removal of the supernatant, the cells were suspended in 1× Annexin binding buffer containing Annexin V‐FITC and PI (100 mg/mL) and exposed to light for 15 min at room temperature. Flow cytometry was conducted using Falcon tip centrifuge tubes on a FACScan flow cytometer (FACSCalibur, BD Pharmingen, USA), and data were analysed using WinMDI 2.9 (BD Biosciences).

### 2.7 Mitochondrial Membrane Potential Assay

Mitochondrial membrane potential (MMP) was determined using an MMP assay kit with JC‐1. Briefly, GBM8401 and U‐87 MG cells were seeded onto 24‐well plates (Orange, UK). After treatment with omaveloxolone (0, 600, 800 and 1000 nM) for 24 h, 10 μg/mL JC‐1 (Sigma, USA) was added to the culture medium at a concentration of 50 μL/well. Subsequently, the cells were incubated at 37°C for 20 min, followed by mitochondrial staining. After washing twice with warm PBS, the cells were fixed with 2% paraformaldehyde and detected using a FACSCalibur flow cytometer (JC‐1). Data were analysed using WinMDI 2.9. JC‐1 was also detected using fluorescence microscopy (Olympus CKX41 and U‐RFLT 50, Japan). MMP was monitored by detecting green (JC‐1 monomer) and red (JC‐1 aggregates) fluorescence intensity under a laser scanning confocal microscope. The ratio of green to red fluorescence intensity was used to represent changes in the MMP of the GBM8401 and U‐87 MG cells.

### 2.8 Western Blotting

Western blotting was performed as previously described. Cell lysates containing 25–40 g of protein were produced on ice. Protein samples were separated using 10% sodium dodecyl sulfate–polyacrylamide gel electrophoresis. After electrophoretic protein separation, proteins were transferred for 2 h onto polyvinylidene difluoride membranes (Millipore, Billerica, MA, USA). Following overnight blocking, the membranes were incubated with primary antibodies against β‐actin for 2 h at room temperature or overnight at 4°C, as described in the Materials and Methods section. After primary antibody incubation and washing, the membranes were incubated for 30–40 min with the appropriate secondary antibody (LI‐COR, Lincoln, NB, USA) at a dilution of 1:20,000, followed by washing with PBS and PBS with Tween 20. After washing, antigens were identified using an Odyssey Near Infrared Fluorescence Imaging System (LI‐COR) or an Enhanced Chemiluminescence Detection Kit (Amersham, Arlington Heights, IL, USA). Finally, the recorded data were normalised to actin following a densitometric analysis, which comprised the integrated densities of the bands, in ImageJ software (NIH, Bethesda, MD, USA).

### 2.9 Immunocytochemistry

GBM8401 and U‐87 MG cells were cultured on biocoated coverslips (BD Biosciences, San Jose, CA, USA) and fixed with 4% paraformaldehyde for 20 min. Subsequently, the coverslips were incubated with primary antibodies overnight at 4°C. The cells were blocked with 10% bovine serum albumin (BSA) at room temperature. A primary antibody against α‐tubulin (1:500, Proteintech, 11224‐1‐AP) was used. After washing with PBS, the cells were incubated with an antirabbit secondary antibody (1:250; Jackson ImmunoResearch Laboratories, PA, USA). Confocal fluorescence images were acquired using a BD CARV II Confocal Imager (NJ, USA) equipped with an oil immersion lens.

### 2.10 Next‐Generation Sequencing

After 24 h of incubation, total RNA was extracted using an RNAzol RT reagent. Subsequently, 1 μg of the extracted RNA was purified using oligo(dT) primers. Magnetic beads were used to capture and fragment eukaryotic mRNA. This fragmented mRNA served as the template for cDNA synthesis. First‐strand cDNA was synthesised using random primers and reverse transcriptase. Subsequently, dNTPs, RNase H and DNA polymerase were added to generate double‐stranded cDNA. Fragment size was determined using an Agilent BioAnalyzer 2100 system. To ensure adequate library quality and size, the concentration of the library was quantified using real‐time PCR. Finally, 150 paired‐end sequencing runs were executed on an Illumina NovaSeq 6000 sequencer. Reads were aligned to the reference genome by using the Counts feature (subread v.2.0.1), and a performance scale table was generated for each gene.

### 2.11 Analysis of Kyoto Encyclopedia of Genes and Genomes Enrichment

To identify key Kyoto Encyclopedia of Genes and Genomes (KEGG) categories, the top 20 pathway maps were analysed using the KEGG database (https://www.genome.jp/kegg/). The names of these KEGG pathways are provided on the vertical axis, and the number of differentially expressed genes in each pathway is presented on the horizontal axis. Each colour indicates the level of significance (adjusted *p* value).

### 2.12 Tissue Microarray Assay

To conduct a tissue microarray assay (TMA), brain primary tumour tissue microarray slides (Cat# GL2082) were obtained from US Biomax (MD, USA). Formalin‐fixed, paraffin‐embedded sections were exposed to deparaffinisation, rehydration and heat‐induced antigen retrieval with 10 mM citrate buffer at pH 6.0. Thereafter, the sections were blocked with 5% BSA and incubated with an antibody against CDC20. To block endogenous peroxidase activity, immunohistochemical staining of CDC20 was performed using a Mouse/Rabbit Probe HRP Labeling kit with a 3,3′‐diaminobenzidine (DAB) Brown reagent (BIOTnA Biotech, Kaohsiung, Taiwan) in accordance with the manufacturer's instructions, followed by counterstaining with haematoxylin.

### 2.13 Three‐Dimensional Tomographic Microscopy

GBM8401 and U‐87 MG cells were seeded onto glass‐bottom dishes with a diameter of 20 mm (Bio‐Rad, Taiwan), followed by incubation for 24 h at 37°C with 5% CO_2_. Subsequently, the morphology of the cells treated with omaveloxolone was analysed under a three‐dimensional (3D) tomographic microscope (3D Cell Explorer Fluo; Nanolive, Taiwan) equipped with a 60× objective. Finally, the morphology of the cells was observed at different time points, and the images (z‐stacks) were processed using STEVE software.

### 2.14 In Vivo Xenograft Model

GBM8401 cells (5 × 10^6^) were harvested and resuspended in 100 μL of PBS. Tumour xenografts were established by injecting GBM8401 cells into the dorsal flank of mice. Male nude mice (aged 3–4 weeks, National Laboratory Animal Center, Taipei, Taiwan) were randomly assigned to each experimental group. After the tumour volume reached 50–100 mm^3^, the mice were intraperitoneally injected twice weekly with different concentrations of omaveloxolone (15, 20 and 25 mg/kg) for 4 weeks or with vehicle (PBS) combined with TMZ for 1 month. Tumour volume was measured every 3 days with callipers and was calculated as *L* × *W* ^2^ × 0.52, where *L* is the length and *W* is the width. Tumour volume and body weight data were recorded until the end of the experiment. All xenograft mouse organs were fixed in 4% neutral buffered formalin, embedded in paraffin, serially cut into 4‐μm sections and stained with haematoxylin and eosin (H&E) for further pathological examination and subsequent immunohistochemical analysis.

### 2.15 Statistical Analysis

The mean ± standard error of the mean (SEM) from at least three independent experiments was statistically analysed using GraphPad software (GraphPad Software, San Diego, CA, USA). For one‐way and two‐way analysis of variance, a *p* threshold of 0.05 was used to determine statistically significant results. In addition, *p < 0.05, **p < 0.01, ***p < 0.005 and ****p < 0.0001 were set to denote significant differences between the control and treatment groups.

## 3 Results

### 3.1 Omaveloxolone Reduced the Viability of GBM Cells in a Time‐ and Dose‐Dependent Manner

**FIGURE 1:**
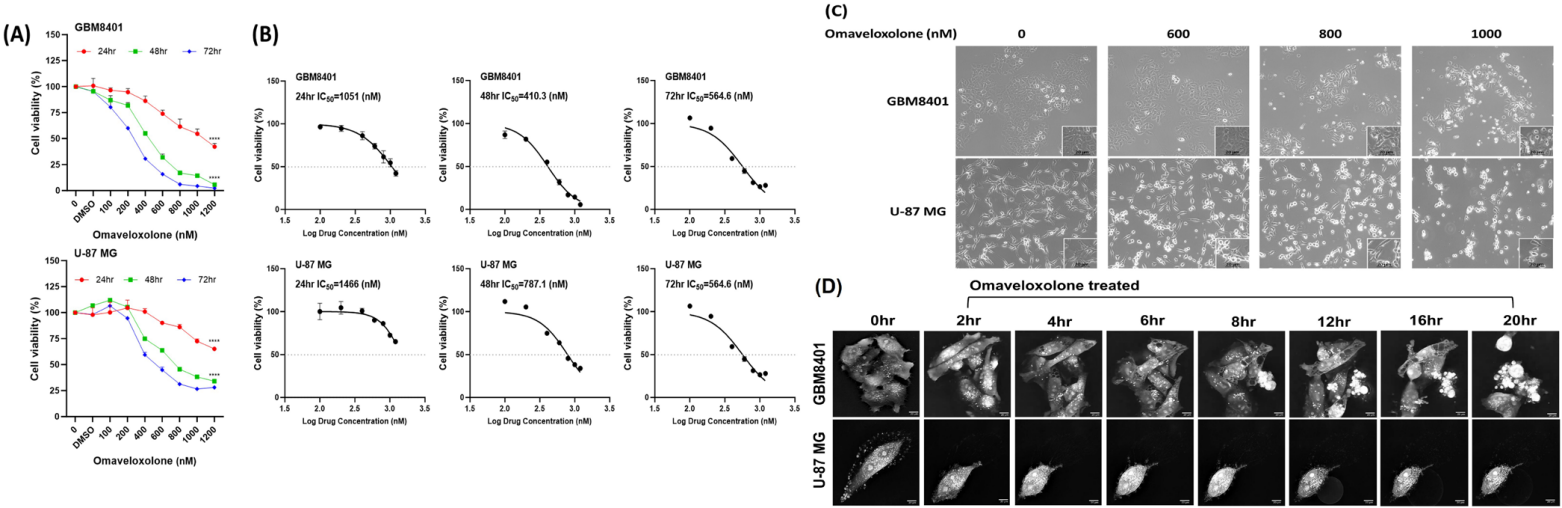
Omaveloxolone inhibited the growth of GBM cells in a time‐ and dose‐dependent manner. (A) Cell viability of omaveloxolone‐treated glioma cells from 24 to 72 h. GBM cells were treated with different concentrations of omaveloxolone (0, DMSO, 100, 200, 400, 600, 800, 1000 and 1200 nM) for 24–72 h. Control cells were exposed to DMSO. Cell viability was measured through a cell viability assay with the PrestoBlue Cell Viability Reagent. (B) The IC_50_ values of omaveloxolone‐treated GBM8401 and U‐87 MG cells at different time points (24 h, 48 h and 72 h). (C) Morphology of GBM8401 and U‐87 MG cells after treatment with omaveloxolone (0, 600, 800 and 1000 nM) for 24 h. (D) Live cell tomographic microscopy images of GBM8401 and U‐87 MG cells treated and untreated with omaveloxolone (800 nM), as observed under a 3D tomographic microscope equipped with a 60× objective. The results are expressed as mean ± standard deviation (SD) of three independent experiments. ****p < 0.00001 compared with the control group (DMSO).

To verify the inhibitory effect of omaveloxolone on GBM cell growth, we conducted a cell viability assay with the PrestoBlue Cell Viability Reagent for omaveloxolone‐treated GBM cells in vitro. We exposed GBM cell lines to different concentrations of omaveloxolone (0, DMSO, 100, 200, 400, 800, 1000 and 1200 nM) for 24–72 h. The results indicated that treatment with omaveloxolone significantly suppressed cell growth in human glioma cells, namely GBM8401 and U‐87 MG cells, in a time‐ and dose‐dependent manner (Figure 1A). In addition, we measured the IC_50_ values of omaveloxolone in GBM8401 cells (24 h = 1051 nM, 48 h = 410.3 nM, and 72 h = 564.6 nM) and U‐87 MG cells (24 h = 1466 nM, 48 h = 787.1 nM, and 72 h = 564.6 nM; Figure 1B). Additionally, omaveloxolone treatment led to notable alterations in cell morphology (Figure 1C). Three‐dimensional tomographic microscopy further confirmed that omaveloxolone induced morphological changes and promoted cell death in a time‐dependent manner (Figure 1D). Collectively, these findings indicate that omaveloxolone effectively reduces GBM cell viability and induces cytotoxic morphological alterations in a concentration‐ and time‐dependent manner.

### 3.2 Omaveloxolone Inhibits Colony Formation and Induces Cell Death in GBM Cells

**FIGURE 2:**
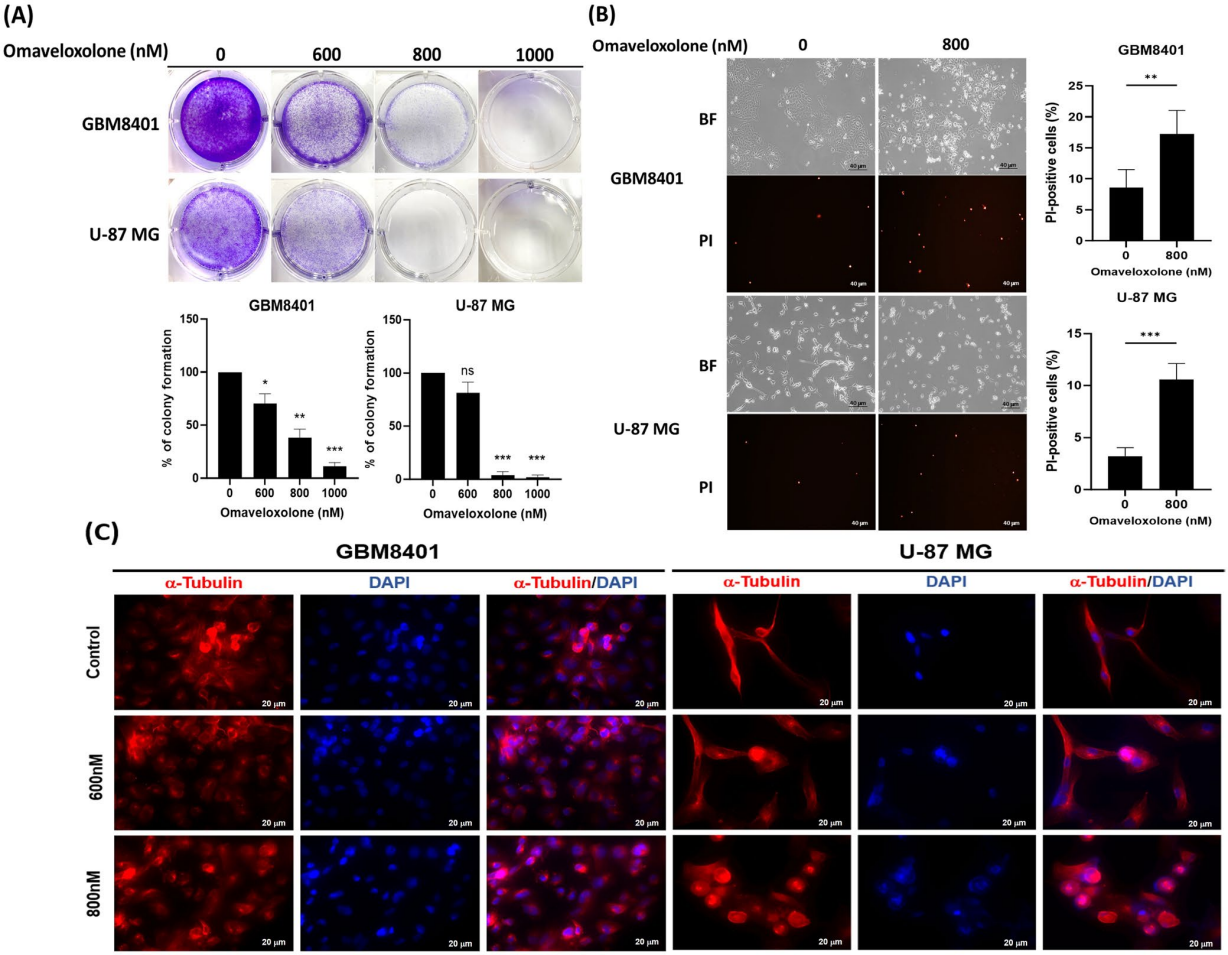
Omaveloxolone reduced the colony formation ability of GBM cells. (A) Colony formation assays of GBM cells treated with omaveloxolone (0, 600, 800 and 1000 nM) for 24 h. These cells were stained with crystal violet. (B) PI staining assay was conducted to examine cell death in GBM cells treated with different concentrations of omaveloxolone for 24 h (BF: Bright field, PI: Propidium iodide). (C) Cells were treated with omaveloxolone (0, 600 and 800 nM), and their nuclei were stained for α‐tubulin (red) and DAPI (blue) under a confocal microscope. Data are expressed as mean ± SD of 3 independent experiments. *p < 0.5, **p < 0.001 and ***p < 0.0001 compared with the control group.

To determine the potential anticancer effects of omaveloxolone, we evaluated the colony formation ability of GBM cells treated with omaveloxolone. We discovered that treatment with omaveloxolone significantly inhibited the growth of GBM8401 and U‐87 MG cells, as evidenced by their reduced colony formation ability (Figure 2A). To examine the effects of omaveloxolone on cell death, we performed PI staining in GBM cells after omaveloxolone treatment. we performed propidium iodide (PI) staining. A substantial increase in PI‐positive cells was observed following omaveloxolone treatment, indicating increased membrane permeability and late‐stage cell death (Figure 2B). Next, we explored the effect of omaveloxolone treatment on microtubule stabilisation in GBM cells and performed the α‐tubulin antibody staining to observe under immunofluorescence microscopy. The results showed that treatment of GBM8401 and U‐87 MG cells with omaveloxolone (0, 600 and 800 nM) for 24 h altered the profile of microtubule (Figure 2C). These results suggest that omaveloxolone not only inhibits GBM cell proliferation but also promotes cell death and alters cytoskeletal integrity.

### 3.3 Omaveloxolone Suppressed the Migration and Invasion of GBM Cells

**FIGURE 3:**
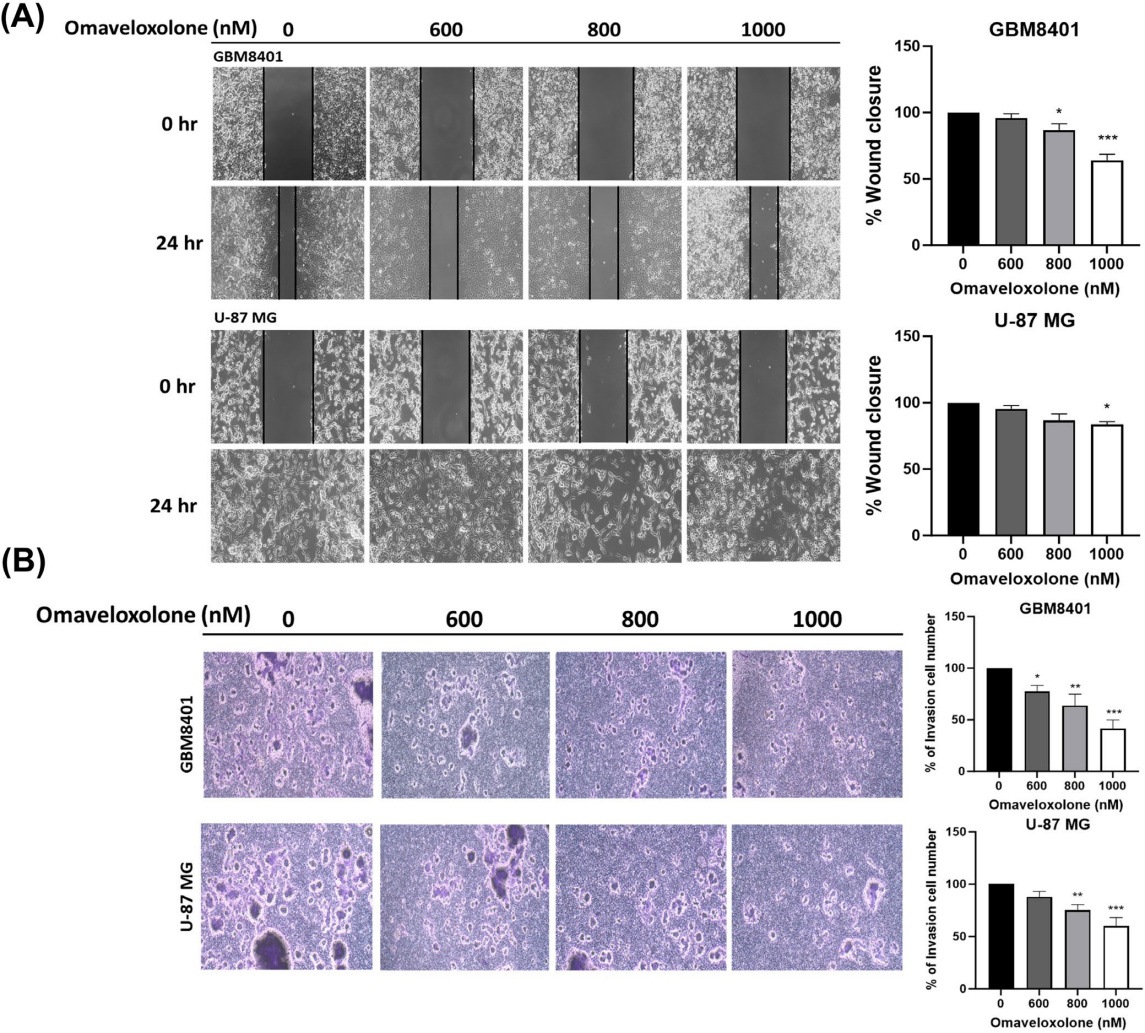
Effect of omaveloxolone on the migration and invasion of glioma cells. (A) Migration ability detected for GBM8401 and U‐87 MG cells treated with omaveloxolone through a wound healing assay. (B) Invasion ability detected for GBM8401 and U‐87 MG cells treated with omaveloxolone through a Transwell invasion assay. Data are expressed as mean ± SD of 3 independent experiments. *p < 0.5, **p < 0.001 and ***p < 0.0001 compared with the control group.

Given that treatment with omaveloxolone suppressed the growth of GBM cells (Figures 1 and 2), we explored whether omaveloxolone affects the migration and invasion of human GBM cells. Wound healing assays showed that omaveloxolone significantly impaired the migratory capacity of both GBM8401 and U‐87 MG cells (Figure 3A). Similarly, Transwell invasion assays demonstrated that omaveloxolone markedly inhibited the invasive potential of both cell lines through Matrigel‐coated membranes (Figure 3B). Together, these findings indicate that omaveloxolone significantly suppresses both the migration and invasion capabilities of GBM cells.

### 3.4 Omaveloxolone Alters the Cell Cycle Distribution of GBM Cells

**FIGURE 4:**
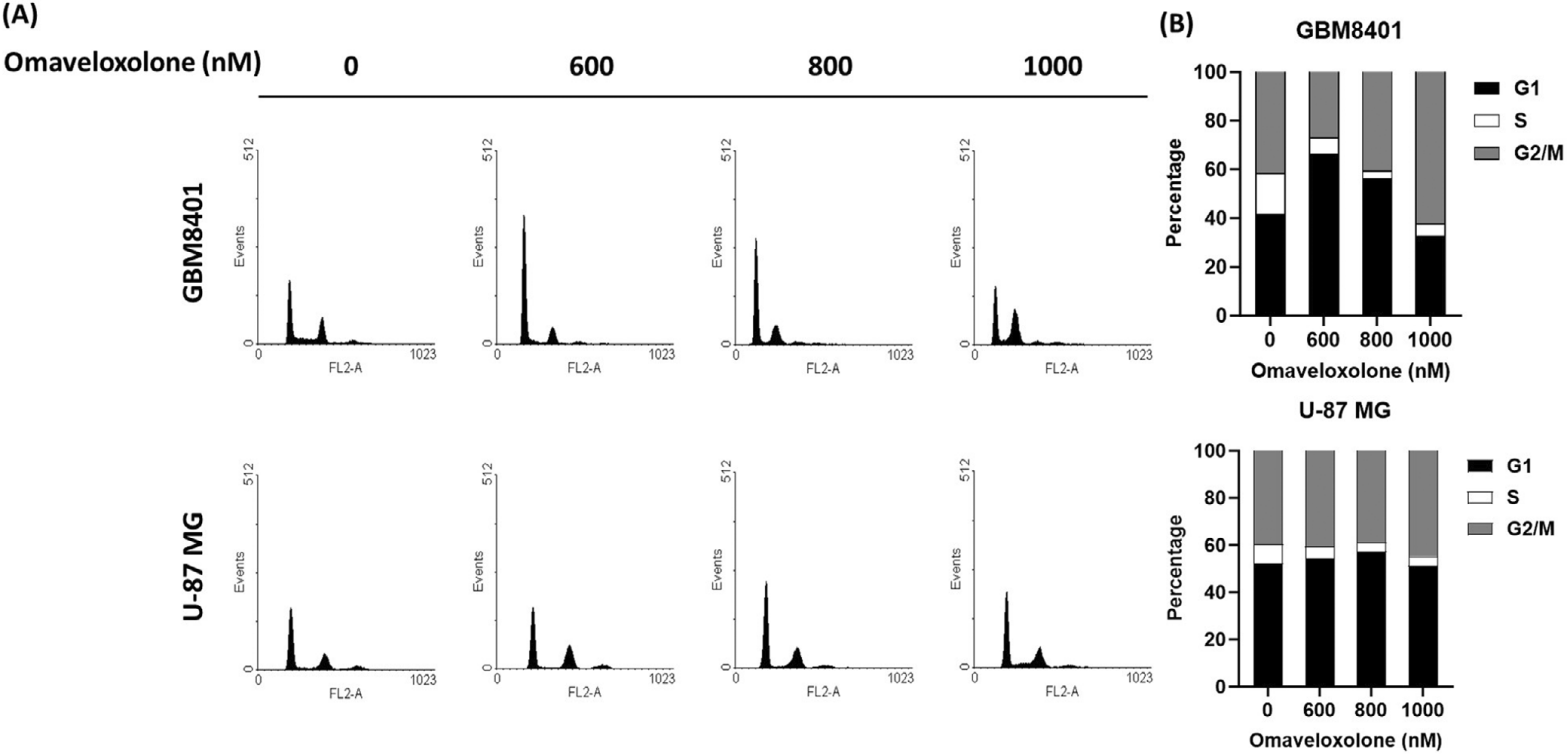
Omaveloxolone regulated the cell cycle phases of GBM cells. (A) Cell cycle analysis of GBM8401 and U‐87 MG cells treated with omaveloxolone (0, 600, 800 and 1000 nM) for 24 h through flow cytometry analysis. (B) Analysis of cell cycle distribution (G1, S and G2/M) in GBM8401 and U‐87 MG cells treated with omaveloxolone (0, 600, 800 and 1000 nM) for 24 h. Cells were stained with PI and analysed using flow cytometry.

To determine whether omaveloxolone‐induced growth inhibition was associated with cell cycle arrest, flow cytometric analysis was performed. We conducted cell cycle analyses to identify the role of omaveloxolone in GBM cells (Figure 4A). The results indicated that treatment with omaveloxolone decreased the percentage of cells in the G0/G1 phase and increased the percentage of cells in the G2/M phase in a dose‐dependent manner (Figure 4B). However, it did not significantly change the cell cycle distribution of U‐87 MG cells. Omaveloxolone changed the cell cycle distribution of glioma cells, particularly GBM8401 cells. These results suggest that omaveloxolone not only inhibits cell growth but also modulates the cell cycle distribution of GBM cells.

### 3.5 Effects of Omaveloxolone on the Apoptosis and Mitochondrial Membrane Potential (MMP) of GBM Cells

**FIGURE 5:**
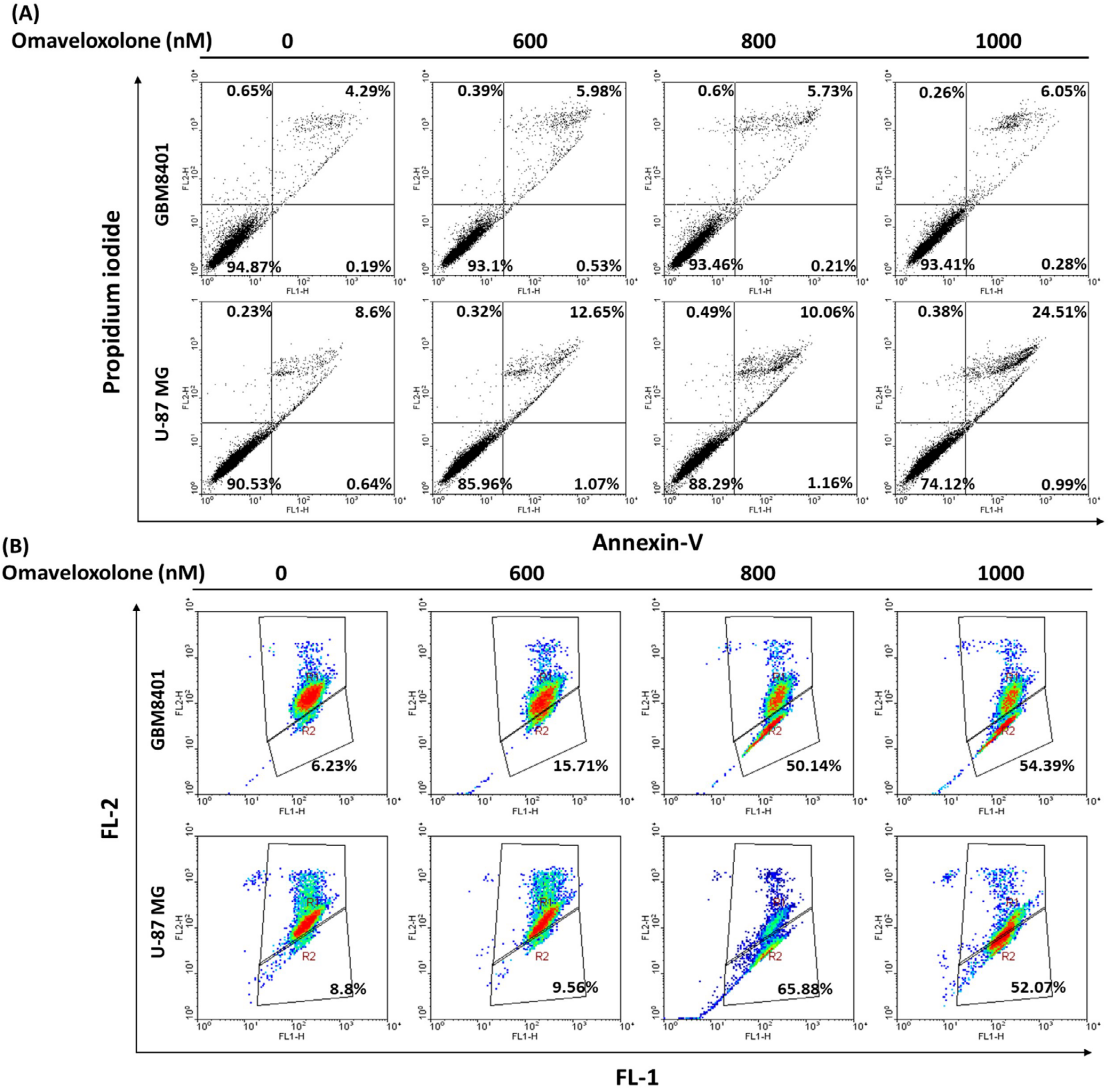
Omaveloxolone increased the apoptosis and regulated the MMP of GBM cells. (A) Representative cell apoptosis of GBM cells treated with omaveloxolone (0, 600, 800 and 1000 nM) for 4 h. Total apoptosis of GBM cells detected by flow cytometry. (B) Representative MMP of GBM cells treated with omaveloxolone (0, 600, 800 and 1000 nM) for 4 h. The data indicate decreased apoptosis in GBM cells treated with omaveloxolone (0, 600, 800 and 1000 nM) with JC‐1 staining.

To verify the effects of omaveloxolone on the apoptosis of GBM cells, we analysed cell apoptosis through Annexin V‐FITC and cell death through PI staining. To explore the potential role of omaveloxolone in the apoptosis of GBM cells, we treated the cells with different concentrations of omaveloxolone (0, 600, 800 and 1000 nM) for 4 h. Subsequently, we harvested the cells and stained them with Annexin V‐FITC and PI through a flow cytometric assay. Flow cytometry revealed that omaveloxolone dose dependently increased the apoptosis of GBM cells (Figure 5A). Notably, treatment of U‐87 MG cells with omaveloxolone increased their cell apoptosis rate from 9.24% to 25.5% (early apoptosis + late apoptosis). MMP loss is a hallmark of apoptosis. To confirm whether apoptosis increased by omaveloxolone can change the MMP of GBM cells, we conducted JC‐1 staining for GBM cells to evaluate their MMP. We also generated FL‐1/FL‐2 dot plots for JC‐1‐stained GBM cells with and without apoptosis. Omaveloxolone‐treated GBM cells with apoptosis, which exhibit green fluorescing monomer expression in the lower area, indicate that they appear apoptotic (600, 800 and 1000 nM omaveloxolone; Figure 5B). Taken together, our data suggest that omaveloxolone increases the apoptosis and changes the MMP of GBM cells.

### 3.6 Modulation of Cell Cycle‐Related Proteins in GBM Cells by Omaveloxolone

**FIGURE 6:**
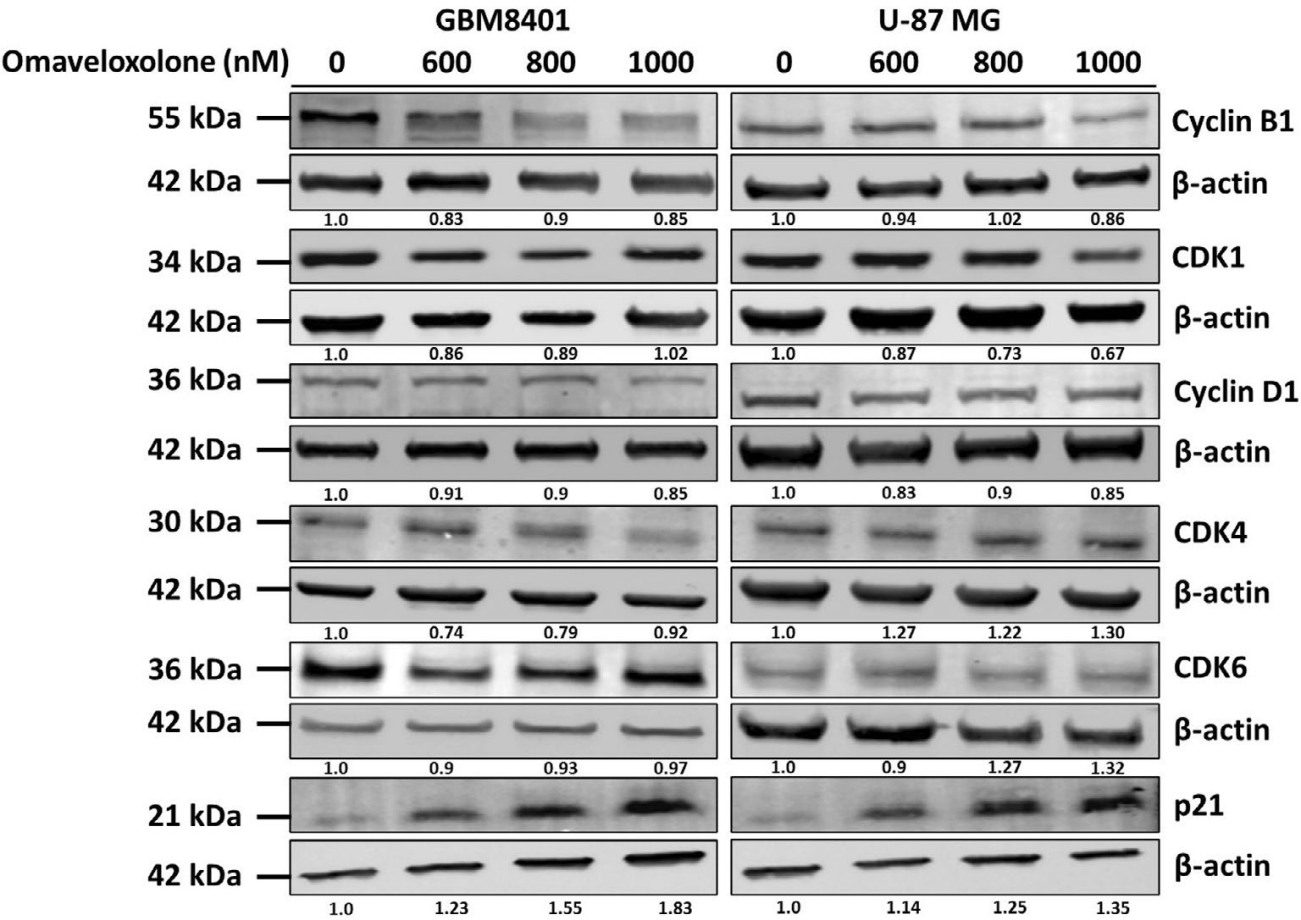
Effect of omaveloxolone on cell cycle‐related protein expression. Omaveloxolone regulated the expression of cell cycle‐related proteins in GBM cells. Both cell types were treated with omaveloxolone (0, 600, 800 and 1000 nM) for 24 h. Subsequently, the pellets were collected, and the lysates were isolated using lysis buffer. The results indicated that treatment with omaveloxolone regulated cell cycle‐related proteins in GBM8401 and U‐87 MG cells. The expression of cyclin D1, CDK4, CDK6, cyclin B1, CDK1 and p21 was analysed using immunoblotting. β‐Actin was used as the loading control.

To determine the molecular mechanism of omaveloxolone‐regulated cell cycle arrest in the G1 to G2/M phase, we examined the expression of various cell cycle‐related proteins through Western blotting. We detected the expression of cell cycle‐related proteins in the G1 phase, including cyclin D1, CDK4 and CDK6. We also detected the expression of cell cycle‐related proteins in the G2/M phase, including cyclin B1 and CDK1. In addition, we detected the expression of p21, a cyclin‐dependent kinase 1 inhibitor. Our data revealed that treatment with omaveloxolone downregulated the expression of cyclin B1, CDK1 and cyclin D1 in GBM cells. In addition, omaveloxolone increased the expression of CDK4 and p21 proteins in GBM8401 and U‐87 MG cells (Figure 6). Taken together, these results indicate that omaveloxolone regulates the expression of cell cycle‐related proteins in GBM cells.

### 3.7 Omaveloxolone Modulated the Expression of Cell Cycle‐Related Genes in GBM Cells

**FIGURE 7:**
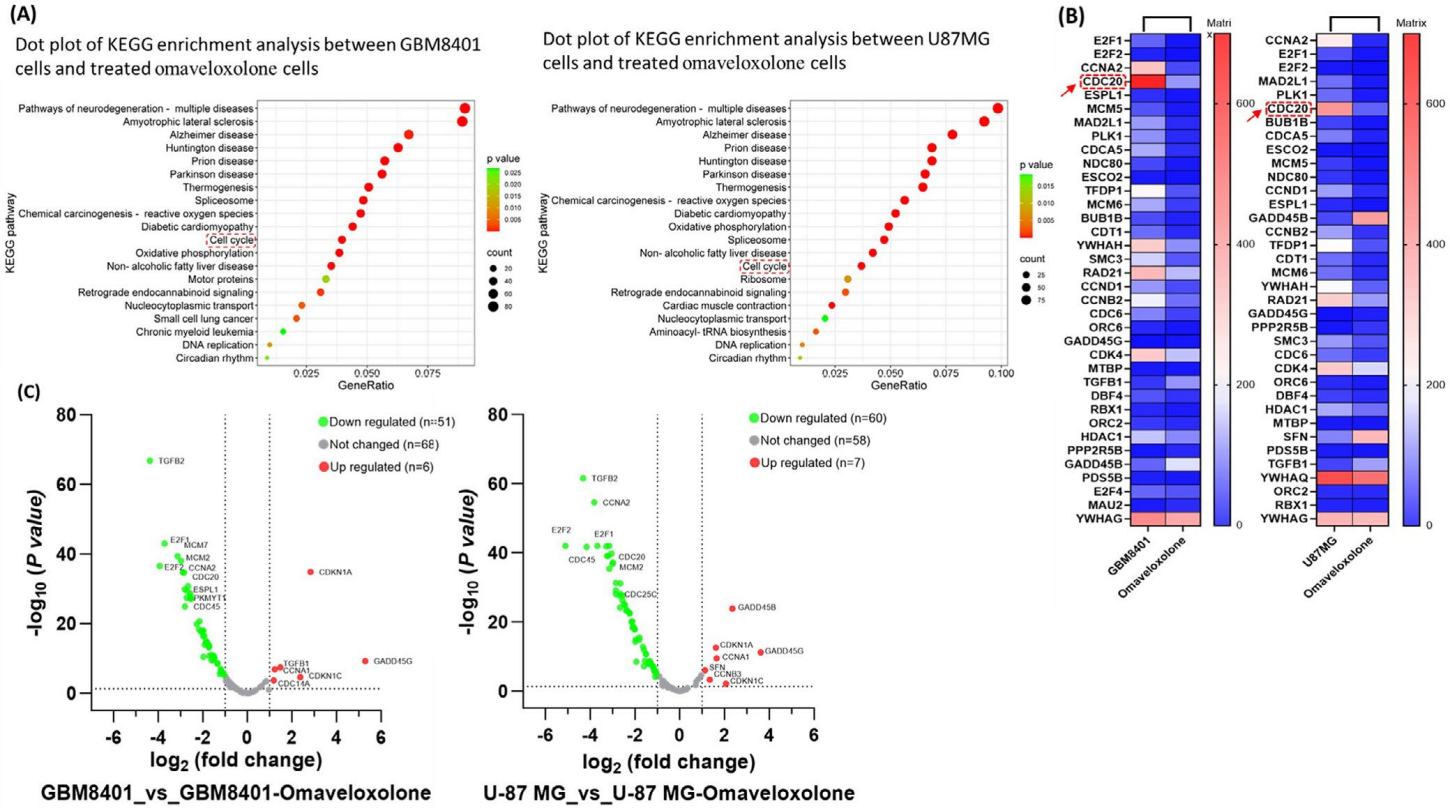
Verification of gene expression in GBM cells treated with omaveloxolone. (A) GeneRatio dot plot of the KEGG pathway analysis of GBM cells treated with omaveloxolone. (B) Heatmap clustering analysis of differential transcripts per million genes following GBM cell treatment with omaveloxolone. (C) Volcano plot of cell cycle‐related gene downregulation or upregulation in GBM cells following treatment with omaveloxolone. Gene log‐fold change (log Fc) expression profile after treatment with omaveloxolone at 800 nM.

We further identified the genes differentially regulated in omaveloxolone‐treated GBM cells with the inhibition of cell proliferation. Omaveloxolone‐treated GBM cells were collected and analysed using RNA sequencing. GBM8401 and U‐87 MG cells were treated with omaveloxolone at 800 nM, and a KEGG pathway enrichment analysis was conducted for these cells. From this KEGG analysis, the dot plot of the top 20 significantly enriched pathways in GBM cells was obtained (Figure 7A). Omaveloxolone regulated the same cell cycle‐related genes in both GBM8401 and U‐87 MG cells, indicating that most of the genes were related to cell cycle signalling pathways. To identify the molecular mechanism through which omaveloxolone regulated the expression of cell cycle‐related genes, we performed RNA sequencing for both treated GBM cells and control cells, and we conducted a heatmap clustering analysis. These analyses revealed 36 cell cycle‐related genes (Figure 7B). A total of 125 cell cycle‐regulated genes were analysed in GBM cells treated with omaveloxolone. Specifically, in the GBM8401 cells, six genes related to the cell cycle were upregulated (fold > 1, *p* < 0.05), and 51 genes were downregulated (fold < −1, *p* < 0.05). In the U‐87 MG cells, seven cell cycle‐related genes were upregulated (fold > 1, *p* < 0.05), and 60 genes were downregulated (fold < −1, *p* < 0.05; Figure 7C). These results suggest that omaveloxolone robustly modulates cell cycle‐associated gene expression, leading to growth inhibition in GBM cells.

### 3.8 *CDC20* Expression Was Associated With Adverse Clinical Outcomes in Patients With GBM

**FIGURE 8:**
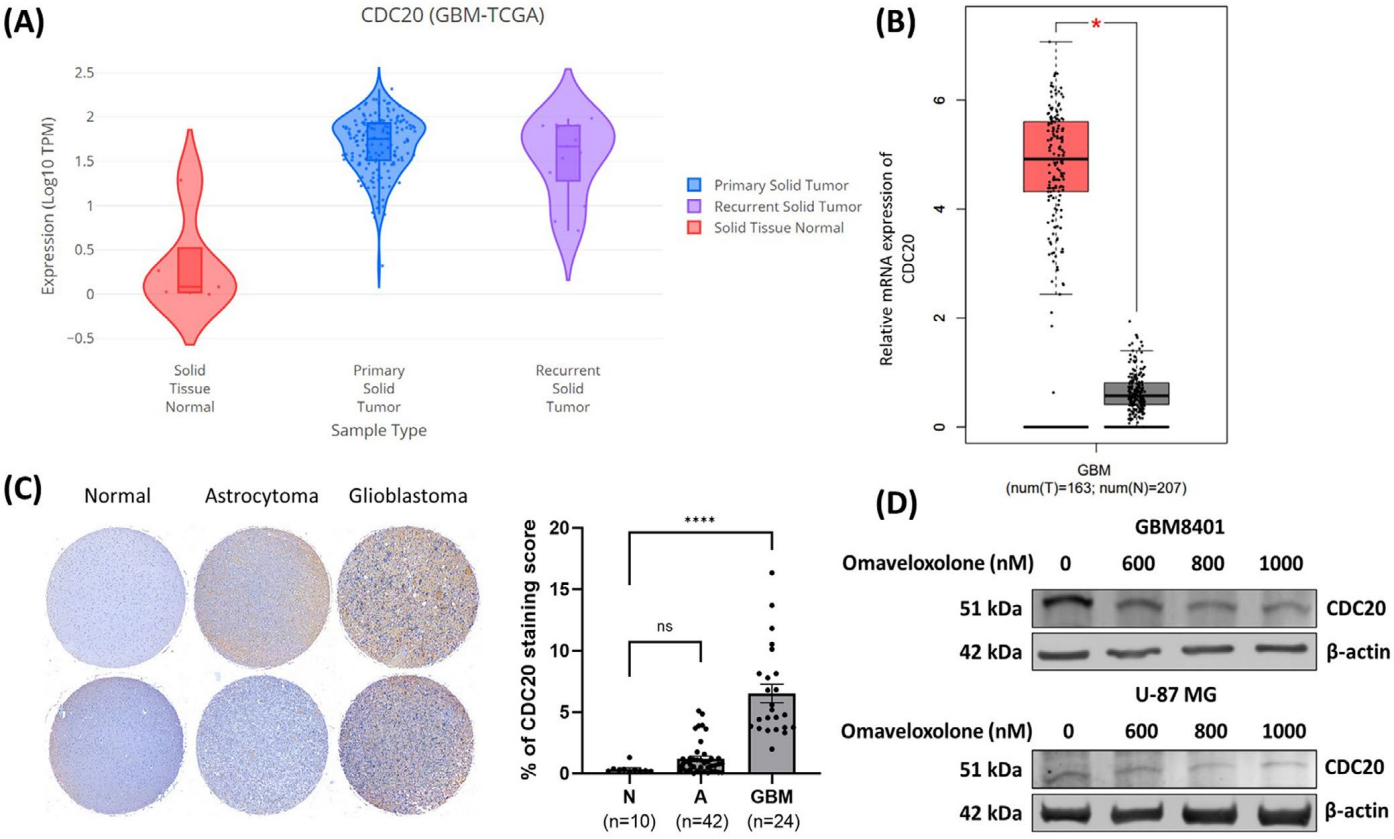
High expression of CDC20 was associated with poor prognosis in patients with GBM. Box plot of the analysis of CDC20 expression in patients with GBM in (A) DriverDBv4 and (B) GEPIA 2 software. (C) Representative TMA data of CDC20 expression in brain cancer (N, normal, *n* = 10; A: Astrocytoma, *n* = 42; GBM, glioblastoma, *n* = 24). (D) Omaveloxolone decreased the expression of CDC20 using immunoblotting. Data are expressed as mean ± SD. *p < 0.05 and ****p < 0.0001. ns, no significant difference.

Given that omaveloxolone regulated the expression of cell cycle‐related genes, we observed that omaveloxolone downregulated the expression of CDC20 in GBM cells (Figure 7). To determine the involvement of *CDC20* gene expression in cancer and whether *CDC20* is a target of omaveloxolone, we analysed the expression of the *CDC20* gene in patients with GBM by using GEPIA 2 (http://gepia2.cancer‐pku.cn/#analysis) and DriverDBv4 (http://driverdb.bioinfomics.org/) software. Analysis of The Cancer Genome Atlas (TCGA) revealed that patients with GBM had higher expression of *CDC20* mRNA compared with those without (Figure 8A,B). Increasing evidence indicates that CDC20 is involved in several biological processes and that many diseases are related to the dysregulation of *CDC20* (8–13). *CDC20* is associated with tumorigenesis and poor prognosis. Its *CDC20* is upregulated in several types of cancer, such as lung cancer, liver cancer and prostate cancer. In this study, to evaluate the association between CDC20 expression and brain cancer grade, we conducted a brain cancer TMA of tissues with different grades of brain cancer, including normal tissues, tissues with astrocytoma and tissues with GBM. The results revealed higher CDC20 expression in the GBM tissues than in the normal or astrocytoma tissues (Figure 8C). After treatment with omaveloxolone, we analysed the levels of the CDC20 protein in GBM cells. The results indicated that omaveloxolone downregulated the expression of CDC20 (Figure 8D). Collectively, these findings suggest that *CDC20* may serve as a critical target of omaveloxolone and that its downregulation may contribute to the antitumor effects observed.

### 3.9 Omaveloxolone Suppressed GBM Cell Proliferation and CDC20 Expression In Vivo

**FIGURE 9:**
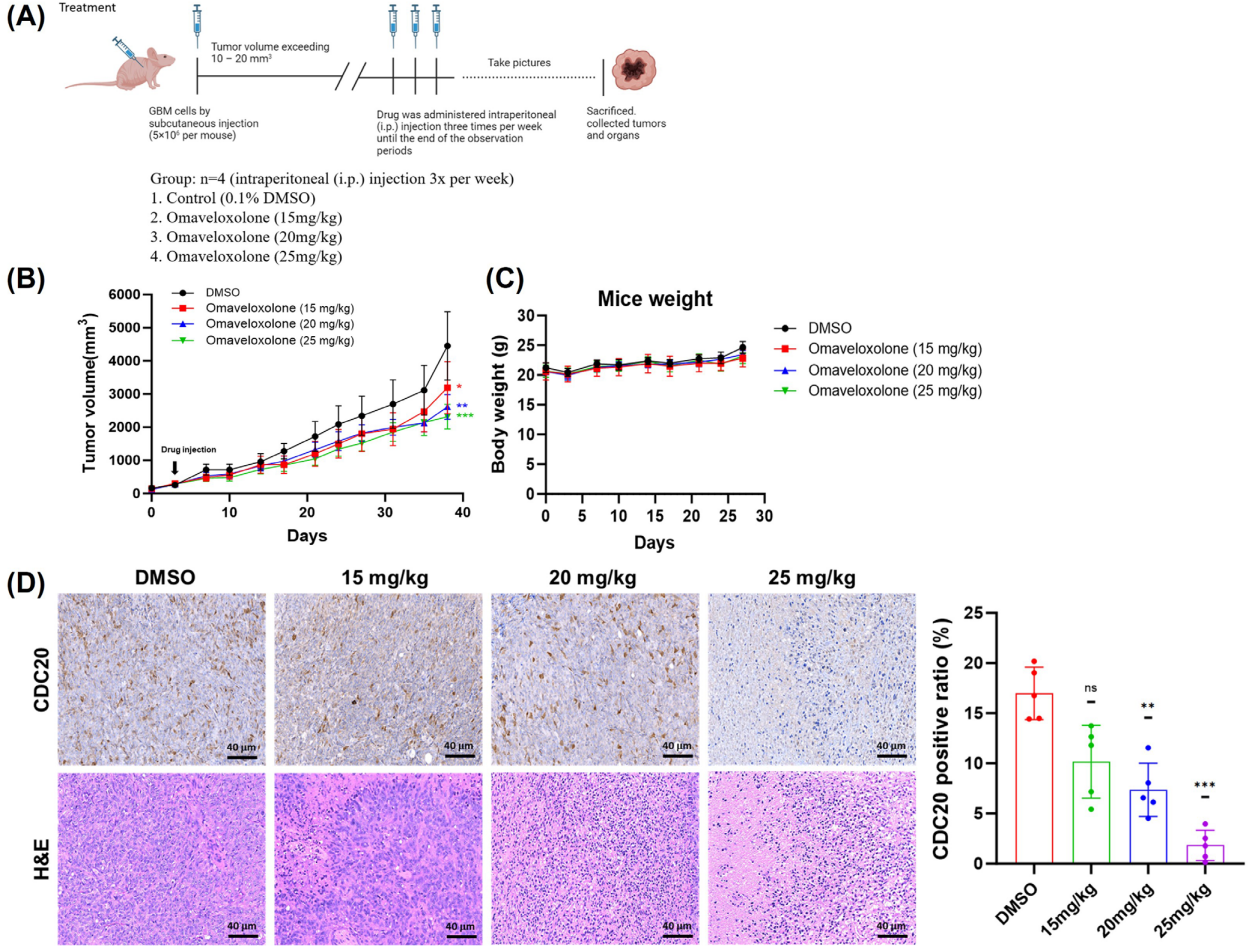
Antitumor activity of omaveloxolone in vivo. (A) Schematic of the experimental subcutaneous GBM8401 tumour model with omaveloxolone treatment. Experiments were conducted using 4‐week‐old male nude mice (BALB/c‐nu). When tumours sizes reached 10–20 mm^3^, an intraperitoneal injection of omaveloxolone (15, 20 or 25 mg/kg, with 0.1% DMSO as control) was administered for 4 weeks. (B) Effect of omaveloxolone tumour volume in mouse xenografts. (C) Effect of omaveloxolone on the body weight of nude mice. (D) CDC20 and H&E‐stained sections from tumours in nude mice treated with omaveloxolone (15, 20 and 25 mg/kg) and the control group. Data are expressed as mean ± SEM (*n* = 5). *p < 0.05, **p < 0.001 and ***p < 0.0001 compared with the control group. This figure was created using BioRender.com.

To determine the potential antitumor protective effects of omaveloxolone in vivo, nude mice were xenografted through subcutaneous inoculation with GBM8401 cells. After 4‐week nude mice (BALB/c‐nu, *n* = 4) received a subcutaneous injection of GBM8401 cells, they received an intraperitoneal injection of omaveloxolone three times per week for 4 weeks (Figure 9A). According to tumour volume measurements, mice with xenogeneic tumours treated with omaveloxolone exhibited greater growth inhibition in a dose‐dependent manner compared with mice with DMSO‐treated tumours (Figure 9B). Notably, treatment with omaveloxolone did not lead to weight loss in the mice (Figure 9C). Consistent with the results of omaveloxolone treatment in GBM cells, treatment with omaveloxolone downregulated the expression of CDC20 (Figure 9D), indicating its effectiveness in inhibiting the growth of GBM cells. Taken together, these findings suggest that omaveloxolone suppresses tumour growth in vivo. We also indicate that omaveloxolone can effectively inhibit the proliferation of GBM cells and can reduce the proportion of CDC20 expression‐positive cells.

## 4 Discussion

In this study, we examined the inhibitory effects of omaveloxolone on the proliferation of GBM8401 and U‐87 MG cells both in vitro and in vivo. Our findings revealed that omaveloxolone increased the proportion of cells in the G2/M phase of the cell cycle, which may be linked to the ability of omaveloxolone to inhibit cell proliferation. In addition, 3D tomographic microscopy revealed that treatment with omaveloxolone altered cell morphology and increased cell death in a time‐dependent manner. Treatment with omaveloxolone also reduced cell migration and invasion, indicating the potential suppression of GBM cell metastasis by omaveloxolone. We further identified the genes differentially regulated by the omaveloxolone‐mediated inhibition of cell proliferation through next‐generation sequencing (NGS). Our findings revealed that omaveloxolone modulated cell cycle‐related genes in both cell lines, leading to the downregulation of CDC20 expression. Furthermore, treatment with omaveloxolone reduced tumour volume and downregulated the expression of CDC20 in vivo, indicating the potential of omaveloxolone as a therapeutic anticancer agent.

Anticancer strategies often involve drugs that induce cell cycle arrest and promote cancer cell apoptosis or autophagy [26]. OA and its derivatives exhibit the aforementioned anticancer effects [27]. Omaveloxolone also exhibits these effects. Overall, our experimental results indicate that omaveloxolone can induce cancer cell apoptosis and cell cycle arrest. Increasing evidence demonstrates that omaveloxolone can induce cell apoptosis in various types of cancer. In the present study, omaveloxolone effectively inhibited GBM cell growth and induced cytotoxicity in vitro. In addition, omaveloxolone significantly changed 3D cell morphology, reduced colony formation, increased cell death, significantly inhibited the migration and invasion of cells and increased the apoptosis and changed the MMP of GBM cells. Thus, omaveloxolone can effectively inhibit the proliferation of GBM cells and reduce the proportion of CDC20 expression‐positive cells both in vivo and in vitro.

Cell cycle regulation is crucial for maintaining the growth of eukaryotic cells. The cell cycle comprises a series of tightly regulated steps; when this regulatory process fails, cancer cells are formed, which divide uncontrollably [28]. A previous study reported that omaveloxolone induced cell cycle arrest [29]. According to the literature, omaveloxolone alters the cell cycle distribution of glioma cells, particularly GBM8401 cells. It regulates the cell cycle and induces cell cycle arrest in several types of cancer, including breast cancer [30], leukaemia [31], neuroblastoma [32] and glioma [33, 34]. In the present study, exposure to omaveloxolone dose‐dependently increased the number of cells in the G2/M phase. This finding suggests that omaveloxolone regulates mitosis and promotes cell death in GBM8401 and U‐87 MG cells. These findings are promising and provide preliminary insights highlighting omaveloxolone as a chemotherapeutic drug candidate.

To ensure appropriate cell cycle progression, the cell cycle progresses through specific checkpoints for cell cycle regulation. These checkpoints are crucial because the cell cycle typically stops in the presence of defects in DNA replication or chromosome segregation, and it progresses once these defects are repaired. The main checkpoints in the cell cycle are the G1 checkpoint [35], the G2/M checkpoint [36] and the spindle checkpoint [37]. The G2/M checkpoint is essential in cellular responses to genotoxic stress, ensuring that cells do not proceed to mitosis until all DNA damage or incomplete replication is adequately repaired, thus maintaining genomic stability [36]. This effect is consistent with previous findings indicating that OA and its derivatives can induce cell cycle arrest in the G2/M phase. For example, CDDO‐imidazolide has been found to induce G2/M cell cycle arrest in BRCA1‐mutated breast cancer cells [30], and CDDO‐Me has been found to significantly arrest K562 cells in the G2/M and S phases [31]. In the present study, omaveloxolone induced cell cycle arrest in the G2/M phase in GBM8401 and U‐87 MG cells. It also downregulated the expression of cyclin B1, CDK1 and cyclin D1 and upregulated the expression of CDK4 and p21 proteins in GBM8401 and U‐87 MG cells, as indicated by our Western blot analysis results. These results suggest that omaveloxolone regulates the cell cycle at the main checkpoints, thereby facilitating cell cycle progression through the S phase and into the G2 phase.

CDC20 is a highly conserved and essential regulator of the cell cycle and is considered a promising therapeutic target in various types of cancer [23]. Higher CDC20 expression has been observed in GBM compared with low‐grade gliomas, with patients with higher‐CDC20‐expressing GBM of the proneural subtype exhibiting significantly shorter overall survival [25]. These findings are corroborated by our data, indicating that the expression of CDC20 is significantly higher in GBM tissues than in normal or astrocytoma tissues (Figure 8). Given the crucial oncogenic role of CDC20 in tumorigenesis, targeting its inhibitors may offer a therapeutic advantage for human cancer. Zeng et al. [38] reported the binding of tosyl‐L‐arginine methyl ester (TAME) to APC, suppressing its activation by modulating the expression of CDC20 and Cdh1. They also indicated that TAME disrupted the binding of CDC20 to APC, thereby inhibiting APC E3 ligase activity. In the present study, NGS analysis revealed that treatment with omaveloxolone downregulated the expression of CDC20 in GBM cells (Figure 8B). Analysis of TCGA revealed that patients with GBM exhibited higher expression of CDC20 mRNA compared with the control group (Figure 8B). Tissue array analysis revealed higher expression of CDC20 in GBM tissues than in normal or astrocytoma tissues (Figure 8C). In addition, omaveloxolone downregulated the expression of CDC20 in vitro (Figure 8D). Taken together, these findings confirm that the expression of CDC20 in GBM cells is associated with malignancy and a poor patient prognosis. In addition, omaveloxolone effectively inhibits the gene and protein expression levels of CDC20 in GBM cells both in vitro and in vivo.

CDC20 plays a crucial role in cell cycle regulation and cancer therapy. Cell growth can be inhibited through three pathways by targeting the regulation of CDC20 and generating the APC/C complex [39]. First, drugs stabilise the APC/CDC20 complex, resulting in mitotic arrest or mitotic slippage, as observed with Apcin. Second, drugs competitively bind to the APC/C core complex, preventing its binding to CDC20, which in turn stabilises CCNB1 and leads to mitotic arrest, as demonstrated by pro‐TAME. Lastly, drugs inhibit the downstream activity of CDC20, causing mitotic arrest, as observed with 9F. Certain natural compounds have been demonstrated to effectively inhibit the expression of CDC20. For example, withaferin A, a bioactive compound isolated from *Withania somnifera* , has been identified to exert anticancer effects through the enhanced degradation of CDC20 [40]. Similarly, NAHA, a N‐alkylated amino acid‐derived sulfonamide hydroxamate, has been found to downregulate the expression of CDC20, leading to the inhibition of cell growth [41]. Ganodermanontriol, a *Ganoderma*‐derived alcohol extracted from medicinal mushrooms, has been shown to decrease the level of CDC20, thereby inhibiting the growth of breast cancer cells [42]. MycoPhyto Complex, a novel mixture of medicinal mushrooms, has been found to hinder cell proliferation and induce cell cycle arrest by targeting CDC20 in breast cancer cells [43]. Genistein, a phytoestrogenic isoflavonoid, has been found to partially exert its tumour‐suppressive effect by inhibiting the expression of CDC20 in GBM, hepatocellular carcinoma and breast cancer cells [44]. Additionally, curcumin has been reported to inhibit the expression of CDC20 in pancreatic cancer cells [45]. Given these findings, we propose that omaveloxolone may be a promising new‐generation inhibitor of CDC20 expression in GBM cells.

Omaveloxolone, an Nrf2 activator, likely induces cell cycle arrest and exerts anticancer effects by modulating the formation of the APC/C‐CDC20 complex. Although preliminary studies suggest that Nrf2 may have a regulatory effect on CDC20, the specific underlying mechanisms and interactions remain to be further elucidated. Nrf2 may influence the function of CDC20 by regulating the expression of cell cycle‐related genes, leading to cell cycle arrest and anticancer effects. Nrf2 is activated under oxidative stress conditions, which can affect the progression of the cell cycle and potentially influence the smooth progression of mitosis. Nrf2 activation can indirectly affect the function of CDC20, influencing cell survival and proliferation. Nrf2 regulates the expression of various genes by binding to ARE, which in turn affects the levels of CDC20. Omaveloxolone regulates cell cycle progression through the G1 phase and G2/M phase and the activity of promoter p21, thereby influencing the formation of the CDK/cyclin complex.

## 5 Conclusion

In this study, we demonstrated that omaveloxolone, a novel synthetic derivative of oleanolic acid, is a promising anticancer agent against human glioma. Omaveloxolone inhibits cell proliferation by downregulating the protein and gene expression of CDC20 in GBM cells in vitro. It also suppresses tumour growth and downregulates the expression of CDC20 in vivo. Further research is required to elucidate the therapeutic potential of omaveloxolone.

## Author Contributions

**Kuan‐Ting Lee:** conceptualization (equal), data curation (equal), methodology (equal), project administration (equal), validation (equal), visualization (equal). **Yi‐Chiang Hsu:** formal analysis (equal), funding acquisition (equal), resources (equal), software (equal), visualization (equal), writing – original draft (equal), writing – review and editing (equal). **Ann‐Shung Lieu:** conceptualization (equal), formal analysis (equal), investigation (equal), project administration (equal), resources (equal), supervision (equal), visualization (equal), writing – review and editing (equal). **Chih‐Lung Lin:** data curation (equal), funding acquisition (equal), methodology (equal), software (equal), validation (equal), writing – original draft (equal). **Tai‐Hsin Tsai:** conceptualization (equal), data curation (equal), formal analysis (equal), funding acquisition (equal), investigation (equal), methodology (equal), resources (equal), software (equal), supervision (equal), validation (equal), visualization (equal), writing – original draft (equal).

## Ethics Statement

All animal studies were conducted in accordance with the Animal Use Protocol and were approved by Chang Gung Memorial Hospital (IACUC‐2023101213).

## Conflicts of Interest

The authors declare no conflicts of interest.

## Data Availability

The data used to support the findings of this study are available in article Supporting Information. All requests for raw data may be sent to the corresponding author.
